# Real-World Survival Outcomes Following Metastasectomy in RAS Wild-Type mCRC: Insights from a Multicentre National Cohort Study

**DOI:** 10.3390/jcm15020467

**Published:** 2026-01-07

**Authors:** İlker Nihat Ökten, Tuba Baydaş, Mahmut Emre Yıldırım, Cemil Bilir, Şuayib Yalçın, Erdem Çubukçu, Eda Tanrıkulu Şimşek, Çağatay Aslan, Faysal Dane, Sinemis Çelik, Ahmet Bilici, Mehmet Ali Nahit Şendur, Bala Başak Öven, Abdurrahman Işıkdoğan, Hacı Mehmet Türk, Mustafa Karaca, Bülent Karabulut, Melike Özçelik, Mahmut Gümüş, Umut Kefeli, Nuri Karadurmuş

**Affiliations:** 1 Department of Medical Oncology, Istanbul Medeniyet University, Göztepe Şehir Hospital, 34722 Istanbul, Türkiye; tuba.baydas@gmail.com (T.B.);; 2 Medical Park Pendik Hospital, 34899 Istanbul, Türkiye; emremahmutyildirim@gmail.com (M.E.Y.); cebilir@yahoo.com (C.B.); 3 Department of Medical Oncology, Faculty of Medicine, Hacettepe University Cancer Institute, 06100 Ankara, Türkiye; suayibyalcin@gmail.com; 4 Uludağ University Hospital, 16059 Bursa, Türkiye; erdemcubukcu@uludag.edu.tr; 5 Anadolu Medical Center, 41400 Kocaeli, Türkiye; edatanrikulu@gmail.com; 6 Department of Medical Oncology, Faculty of Medicine, Izmir Ekonomi University, Medical Park Hospital, 35330 Izmir, Türkiye; arslancagatay@yahoo.com; 7 Acıbadem Altunizade Hospital, 34662 Istanbul, Türkiye; faysaldane@yahoo.com; 8 Istanbul Oncology Hospital, 34846 Istanbul, Türkiye; drsinemiscelik@gmail.com; 9 Istanbul Medipol University Hospital, 34214 Istanbul, Türkiye; ahmet.bilici@medipol.com.tr; 10 Department of Medical Oncology, Ankara Bilkent City Hospital, 06800 Ankara, Türkiye; 11 Istanbul Yeditepe University Hospital, 34755 Istanbul, Türkiye; basakoven@yahoo.com; 12 Dicle University Hospital, 21280 Diyarbakır, Türkiye; 13 Bezmialem Vakıf University Hospital, 34093 Istanbul, Türkiye; 14 Department of Medical Oncology, Akdeniz University School of Medicine, 07070 Antalya, Türkiye; 15 Acıbadem Kent Hospital, 35620 Izmir, Türkiye; 16 Departmant of Medical Oncology, Umraniye Education and Research Hospital, 34764 Istanbul, Türkiye; 17 Department of Oncology, Faculty of Medicine, Kocaeli University, 41001 Kocaeli, Türkiye; 18 Department of Medical Oncology, University of Health Sciences, Gülhane Training and Research Hospital, 06010 Ankara, Türkiye

**Keywords:** metastatic colorectal cancer, metastasectomy, RAS wild-type, real-world data, survival outcomes, CA19-9

## Abstract

**Background**: Metastasectomy is a cornerstone of multimodal management in metastatic colorectal cancer (mCRC), yet contemporary real-world data focusing specifically on RAS wild-type (RAS-WT) disease remain limited. We aimed to evaluate survival outcomes and prognostic factors associated with metastasectomy in patients with RAS-WT mCRC using a large national multicentre registry. **Methods**: This retrospective cohort study utilized data from the ONKO-KOLON Türkiye registry. A total of 1079 patients with pathologically confirmed KRAS/NRAS wild-type mCRC were identified and categorized according to receipt of metastasectomy. Overall survival (OS) and progression-free survival (PFS) were estimated using the Kaplan–Meier method and compared with log-rank tests across multiple clinically relevant time origins, including metastatic diagnosis, initial colorectal cancer diagnosis, and time of metastasectomy. Prognostic factors within the metastasectomy cohort were assessed using univariate Cox proportional hazards models. Serum CEA and CA19-9 were analyzed after log_10_ transformation. **Results**: Among 1079 patients, 185 (17.1%) underwent metastasectomy. Patients receiving metastasectomy demonstrated significantly longer OS compared with those managed non-surgically when survival was calculated from the time of metastatic diagnosis (hazard ratio [HR] for death 0.36, 95% CI 0.27–0.47; *p* < 0.001), as well as improved PFS (HR for progression or death 0.39, 95% CI 0.30–0.52; *p* < 0.001). The survival advantage remained consistent when OS was measured from the time of initial colorectal cancer diagnosis (HR 0.37, 95% CI 0.25–0.50; *p* < 0.001). Median OS following metastasectomy was 43 months (95% CI 31.4–45.6). In univariate analyses within the metastasectomy cohort, higher baseline CA19-9 levels were significantly associated with inferior OS when analyzed both as a continuous variable (per log_10_ increase; HR 1.81, 95% CI 1.20–2.75; *p* = 0.005) and as a categorical variable (above vs. below threshold; HR 0.37, 95% CI 0.16–0.86; *p* = 0.021). Other clinicopathologic factors, including age, CEA, tumor sidedness, grade, MSI status, and metastatic burden, were not significantly associated with survival. **Conclusions**: In this large, real-world national cohort of RAS-WT mCRC, metastasectomy was strongly associated with prolonged survival across multiple clinically relevant time frames. Within surgically treated patients, baseline CA19-9 emerged as the most informative prognostic marker, while traditional clinicopathologic variables showed limited discriminatory value. These findings highlight the importance of careful patient selection and support further prospective studies integrating molecular and biomarker-based strategies to refine prognostication and optimize surgical decision-making in RAS-WT mCRC.

## 1. Introduction

Colorectal cancer (CRC) remains a major global health challenge, with more than half of patients developing metastatic disease during the course of illness, most commonly to the liver due to both portal venous drainage patterns and the unique immunometabolic characteristics of the hepatic microenvironment [1]. Surgical resection of colorectal liver metastases (CRLM) has long been established as the most effective curative-intent strategy, offering 5-year overall survival rates approaching 40–60% in appropriately selected patients—substantially superior to outcomes achieved with systemic therapy alone [2,3,4,5].

Advances in systemic therapy over the past two decades have dramatically expanded the population eligible for metastasectomy. The introduction of oxaliplatin- and irinotecan-based chemotherapy, combined with biologic agents, has significantly improved radiologic response rates and enabled conversion of previously unresectable disease to resectable states in a meaningful proportion of patients [6,7,8]. Among these biologic agents, anti-EGFR therapy has played a significant role in the management of RAS wild-type metastatic CRC (mCRC). Randomized trials have consistently shown that cetuximab or panitumumab added to chemotherapy improves objective response rates and enhances secondary resectability in RAS-WT and left-sided tumors, underscoring the biological sensitivity of this molecular subgroup to epidermal growth factor receptor inhibition [9,10,11,12].

The prognostic significance of RAS mutational status has been firmly established, with numerous studies demonstrating inferior post-resection outcomes in RAS-mutant tumors when compared with their wild-type counterparts [13,14,15]. As a result, RAS-WT disease is now widely regarded as the subgroup most likely to achieve durable benefit from aggressive multimodal management, including metastasectomy. In parallel, contemporary translational research has provided deeper insight into the metastatic process, revealing that liver colonization is shaped by tumor–microenvironment interactions, stromal remodeling, and immune modulation—features that help explain the biological heterogeneity observed among patients with ostensibly similar clinicopathologic profiles [1].

Despite these advances, real-world evidence describing outcomes of metastasectomy exclusively in RAS-WT populations remains limited, and most existing prognostic models were developed in eras preceding modern biologic therapy and molecular stratification [13,14,15]. Additionally, data from Türkiye have highlighted the feasibility and survival benefit of CRLM resection across diverse institutional settings, including analyses illustrating the effectiveness of liver- and lung-directed interventions within national cohorts [16,17].

Given the increasing global and regional emphasis on precision selection for metastasectomy, there is a critical need for large, multicentre, real-world datasets that evaluate survival outcomes and prognostic factors specifically within RAS-WT mCRC. The ONKO-KOLON Türkiye registry provides a unique opportunity to address this gap by capturing detailed clinical, molecular, and treatment data across multiple centers in a real-world national population. Therefore, in this study, we aimed to (1) characterize the clinicopathologic and treatment features of RAS-WT mCRC patients undergoing metastasectomy, (2) evaluate overall survival outcomes in this KRAS wild-type cohort, and (3) identify prognostic variables influencing survival in real-world practice. By integrating contemporary systemic therapy patterns with national-level surgical data, this study seeks to clarify the modern role of metastasectomy in RAS-WT mCRC.

## 2. Methods and Materials

### 2.1. Study Design and Population

This study was derived from the ONKO-KOLON Türkiye registry, a nationwide, real-world database that prospectively collects demographic, clinicopathologic, and treatment information of patients diagnosed with metastatic colorectal cancer across 28 participating oncology centers in Türkiye. A total of 1079 patients with pathologically confirmed KRAS, NRAS wild-type metastatic colorectal cancer were identified in the registry. For the present analysis, patients were classified according to whether they underwent metastasectomy at any time during their disease course. Metastasectomy was defined as any surgical intervention aimed at the complete or partial removal of metastatic lesions for either curative or cytoreductive intent.

**Figure 1:**
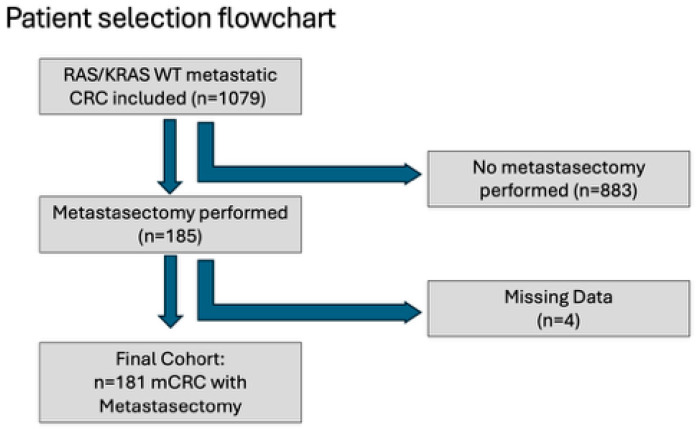
Patient Selection Flowchart.

Among the entire cohort, 185 patients had undergone at least one metastasectomy. Survival outcomes could be reliably assessed in 181 of these patients; four cases were excluded due to inconsistent or missing dates. These 181 patients comprised the analytic metastasectomy cohort. For comparative analyses, baseline demographic and clinicopathologic characteristics of patients with and without metastasectomy were evaluated using the full registry dataset (Figure 1).

### 2.2. Data Collection and Variables

Extracted variables included age at diagnosis, sex, smoking status, primary tumor location and sidedness, histologic grade, presence of a mucinous component, microsatellite instability (MSI) status, BRAF mutation status, baseline serum carcinoembryonic antigen (CEA) and carbohydrate antigen 19-9 (CA19-9) levels, number and sites of metastatic organs, timing and site of metastasectomy, and the number of systemic therapy lines received. Overall survival (OS) was evaluated using predefined time origins to ensure methodological transparency and comparability across analyses, including from the date of initial colorectal cancer diagnosis, from the date of metastatic disease diagnosis, and from the date of metastasectomy for patients undergoing surgical resection. For all OS analyses, survival time was calculated until death from any cause or last known follow-up. Progression-free survival (PFS) was defined as the time from the date of metastatic disease diagnosis to the first documented radiologic or clinical disease progression or death from any cause, whichever occurred first, with disease progression assessed according to routine clinical and imaging follow-up as recorded in the registry. Survival status and follow-up dates were obtained from the ONKO-KOLON registry, routine clinical visits, and national death notification systems.

### 2.3. Statistical Analysis

Baseline characteristics of the metastasectomy and non-metastasectomy groups were compared using χ^2^ tests for categorical variables and Student’s *t*-test or Mann–Whitney *U* test for continuous variables, as appropriate based on data distribution. Survival outcomes were estimated using the Kaplan–Meier method, and survival curves were compared using the log-rank test. Median overall survival (OS), progression-free survival (PFS), 12-, 24-, and 36-month survival probabilities, and corresponding 95% confidence intervals (CIs) were calculated.

In the metastasectomy subgroup, potential prognostic factors for overall survival were evaluated using univariate Cox proportional hazards models. Variables assessed included demographic, clinicopathologic, metastatic, and laboratory parameters. Serum CEA and CA19-9 levels were analyzed as continuous variables after log_10_ transformation to account for right-skewed distributions.

Multivariable Cox regression modeling was explored; however, due to the limited number of death events, substantial missingness across several covariates, and resulting model instability and non-convergence, a final multivariable model was not retained. To avoid overfitting and unreliable estimates, results for the metastasectomy subgroup are therefore presented as univariate associations.

All Cox regression analyses were conducted using complete-case analysis for the variables included in each model; no imputation was performed for missing data. Statistical analyses were performed using Python 3.8 (pandas, numpy, and survival analysis routines equivalent to the lifelines package) and cross-validated against SPSS version 26.0.

## 3. Results

In total, 1079 patients were included, comprising 185 patients in the metastasectomy cohort and 894 patients in the non-metastasectomy cohort (Table 1). Median age (62.0 vs. 60.5 years, *p* = 0.084) and sex distribution (*p* = 0.799) were comparable between groups. Patients who underwent metastasectomy were significantly more likely to have liver metastases as the dominant site (84.9% vs. 67.4%, *p* = 0.001) and to present with liver-only metastatic disease (55.1% vs. 35.9%, *p* < 0.001). Significant differences were also observed in histological grade, presence of a mucinous component, and MSI status between the two cohorts (all *p* < 0.001). Baseline tumor marker levels differed between groups, with lower median CEA (32.0 vs. 77.0 ng/mL, *p* < 0.001) and CA19-9 levels (25.0 vs. 38.5 U/mL, *p* = 0.047) in the metastasectomy cohort. ECOG performance status, smoking status, primary tumor location, number of metastatic organs, number of systemic therapy lines received, and BRAF mutation status did not significantly differ between cohorts (all *p* > 0.05) (Table 1).

### 3.1. Metastasectomy Cohort Characteristics

Among the 185 patients who underwent metastasectomy, the majority presented with synchronous metastatic disease (*n* = 136, 73.5%), while 49 patients (26.5%) had metachronous metastases. At the time of metastatic diagnosis, the liver was the predominant site of metastasis (84.9%), followed by peritoneal (5.4%), lung (3.2%), lymph node (3.2%), and, less frequently, adrenal, brain, or other sites.

With respect to surgical timing, metastasectomy was most commonly performed in a synchronous or upfront setting (69.2%), either as de novo resection or synchronous removal of metastatic lesions. Metachronous metastasectomy following primary tumor surgery was performed in 26.5% of patients, while a smaller subset (4.3%) underwent metastasectomy after receiving systemic therapy.

The liver was the most frequent site of metastasectomy (78.4%), whereas lung, lymph node, brain, bone, and adrenal resections were less common. At the time of metastasectomy, most patients had involvement of a single metastatic organ (67.0%), while 27.0% had two metastatic organs and fewer than 6% had three or more involved organs (Table 2).

### 3.2. Survival Outcomes

Kaplan–Meier analyses demonstrated consistent and clinically meaningful differences in survival outcomes according to metastasectomy status across multiple time origins. When overall survival was calculated from the time of metastatic disease diagnosis, patients who underwent metastasectomy experienced significantly longer survival compared with those managed without surgical resection (hazard ratio [HR] for death 0.36, 95% CI 0.27–0.47; *p* < 0.001). Similarly, progression-free survival measured from the time of metastatic diagnosis was significantly prolonged in the metastasectomy cohort (HR for progression or death 0.39, 95% CI 0.30–0.52; *p* < 0.001).

To provide a population-level context, overall survival was additionally evaluated from the time of initial colorectal cancer diagnosis. In this analysis, metastasectomy remained strongly associated with improved survival outcomes, with a substantially lower risk of death compared with non-metastasectomy patients (HR 0.37, 95% CI 0.25–0.50; *p* < 0.001).

**Figure 2:**
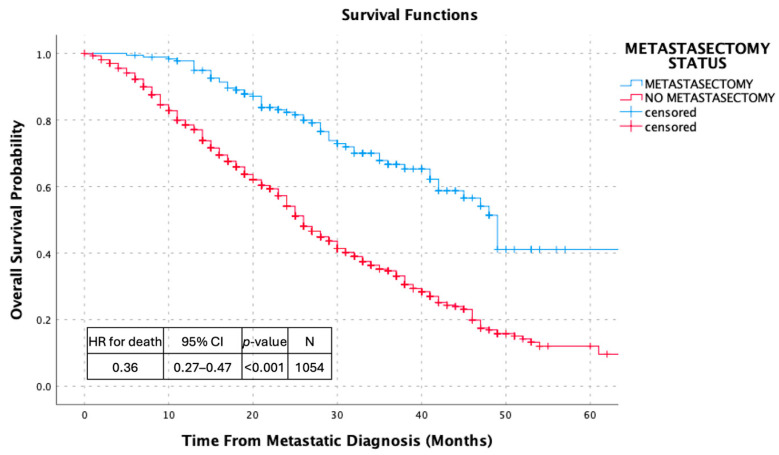
Kaplan–Meier Overall Survival Curves Comparing Patients With and Without Metastasectomy.

**Figure 3:**
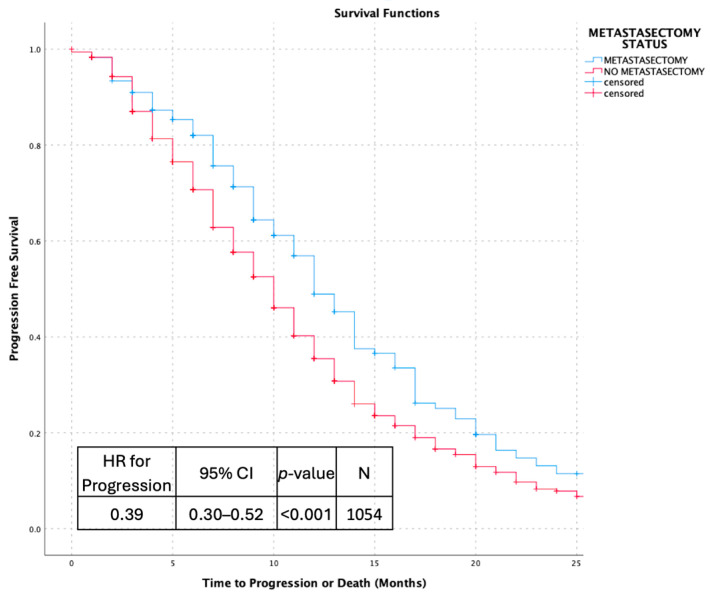
Kaplan–Meier Progression Free Survival Curves Comparing Patients With and Without Metastasectomy.

**Figure 4:**
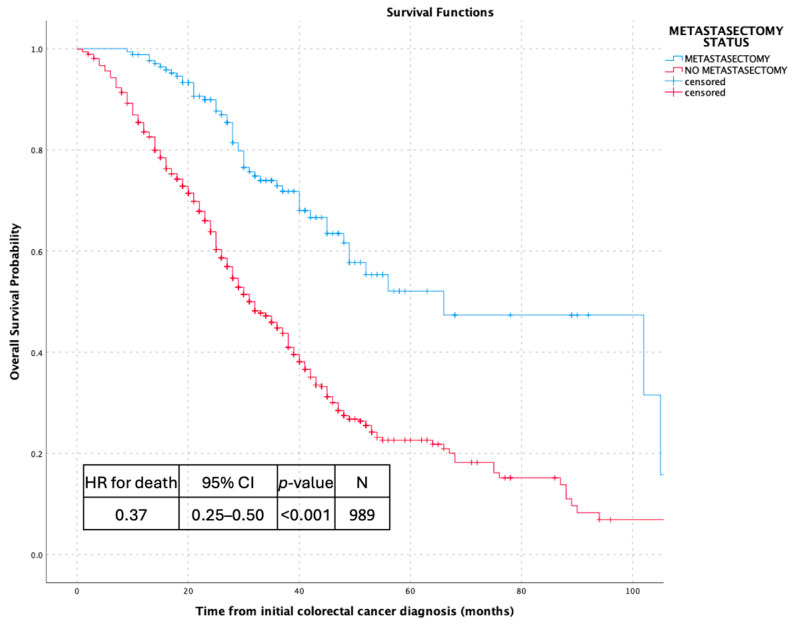
Overall survival from initial colorectal cancer diagnosis according to metastasectomy status.

**Figure 5:**
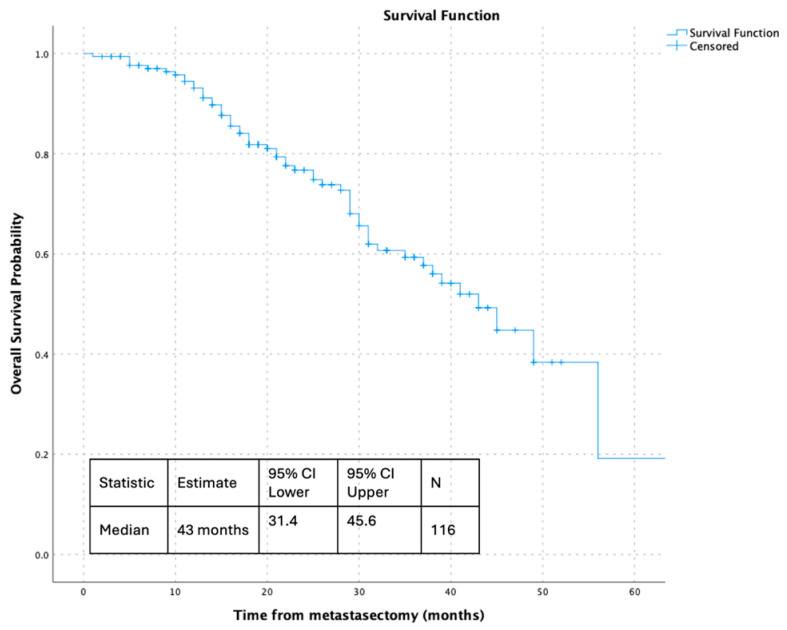
Overall survival following metastasectomy in the surgically treated cohort.

Within the surgically treated cohort, overall survival measured from the time of metastasectomy demonstrated durable post-operative outcomes. The median overall survival following metastasectomy was 43 months (95% CI 31.4–45.6), highlighting prolonged survival after surgical management of metastatic disease. Collectively, these analyses illustrate the association between metastasectomy and favorable survival outcomes across distinct and clinically relevant time origins, as shown in Figure 2, Figure 3, Figure 4 and Figure 5.

### 3.3. Univariate Analysis

Univariate Cox proportional hazards analyses were performed to identify prognostic factors associated with overall survival among patients who underwent metastasectomy. Among the evaluated variables, baseline CA19-9 demonstrated the strongest prognostic impact. Higher baseline CA19-9 levels, analyzed both as a continuous variable (per 1 log_10_ increase) and as a categorical variable (above vs. below threshold), were significantly associated with inferior overall survival (HR 1.81, 95% CI 1.20–2.75, *p* = 0.005; and HR 0.37, 95% CI 0.16–0.86, *p* = 0.021, respectively).

Sex showed a borderline association with survival, with female patients exhibiting a trend toward improved outcomes compared with male patients (HR 0.57, 95% CI 0.32–1.02, *p* = 0.059). Histologic grade also demonstrated a non-significant trend toward worse survival for grade 3 tumors compared with grade 1–2 disease (HR 3.16, 95% CI 0.75–13.37, *p* = 0.118).

**Figure 6:**
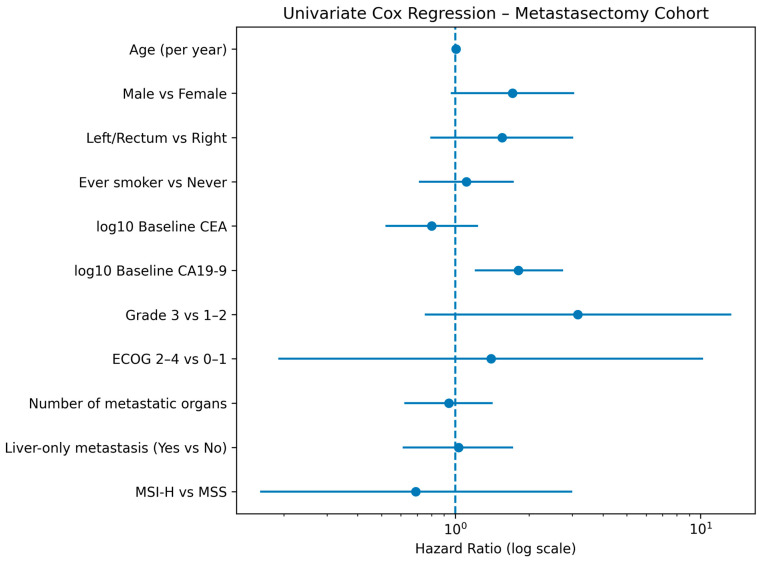
Forest Plot of Univariate Cox Regression Results for Predictors of Overall Survival in the Metastasectomy Cohort.

Other clinical and pathological variables, including age, smoking status, ECOG performance status, number of metastatic organs, liver-only metastatic disease, primary tumor sidedness, mucinous histology, MSI status, and baseline CEA (analyzed as both continuous and categorical variables), were not significantly associated with overall survival in univariate analyses. Detailed hazard ratios, confidence intervals, event counts, and numbers of patients included for each variable are summarized in Table 3, Figure 6.

Given the limited number of death events within the metastasectomy subgroup and the substantial proportion of missing data for several clinicopathologic variables, multivariable Cox regression modeling was explored but resulted in unstable estimates and model non-convergence. In univariate analyses, only baseline CA19-9 remained significantly associated with overall survival. Therefore, to avoid overfitting and misinterpretation, no multivariable Cox model was retained for this subgroup, and results are presented as univariate associations.

Taken together, while log10 CA19-9 emerged as the only statistically significant predictor of overall survival, the trends observed for female sex, left-sided/rectal primary tumors, and non-high-grade histology suggest that these clinicopathologic factors may also contribute meaningfully to prognosis within the metastasectomy cohort, despite not reaching conventional thresholds for statistical significance.

## 4. Discussion

In this multicentre, real-world registry of RAS-wild-type metastatic colorectal cancer, metastasectomy was associated with substantially longer overall survival compared with non-surgical management. Patients who underwent metastasectomy had longer median OS and consistently higher 1-, 2-, and 3-year survival probabilities than those treated without surgery, with early and persistent separation of the Kaplan–Meier curves and a significantly reduced hazard of death in the metastasectomy group. These findings demonstrate a strong association between metastasectomy and favorable survival outcomes, consistent with historical surgical series reporting 5-year OS rates of approximately 40–60% in carefully selected patients [2,3,4,5,18,19].

Our data also highlight the extent to which patients referred for metastasectomy represent a biologically and clinically favourable subset. Compared with patients who did not undergo resection, the metastasectomy cohort more frequently had liver-predominant disease, lower baseline CEA and CA19-9 levels, and a higher proportion of low-grade tumors, while BRAF status and ECOG performance were broadly similar between groups. These differences reflect real-world selection processes rather than treatment effects per se, and mirror prior studies showing that metastasectomy candidates typically have limited metastatic burden, favourable tumor biology, and resectable anatomy [13,14,15,20,21]. Accordingly, the observed survival advantage should be interpreted as an association within a selected population rather than as evidence of a direct causal effect of surgery alone.

When focusing on the metastasectomy subgroup, several conventional clinicopathologic variables showed limited prognostic impact. Age, mucinous histology, MSI status, and baseline CEA were not significantly associated with overall survival, while sex and primary tumor sidedness demonstrated only borderline associations in univariate analyses. These findings suggest that once patients are selected for surgical management, traditional clinicopathologic factors may have reduced discriminatory power, potentially reflecting the homogenizing effect of patient selection and contemporary systemic therapies [13,14,15,22].

In univariate Cox analyses, baseline CA19-9—analyzed after log_10_ transformation—emerged as the only variable significantly associated with mortality in the metastasectomy cohort. Although sex, sidedness, and grade met a liberal inclusion threshold and were explored in multivariable models, model stability was limited by event numbers and variable missingness, resulting in attenuation of effects for all variables except CA19-9. Several studies have similarly identified CA19-9 as a prognostic biomarker in colorectal liver metastasis, correlating with tumor burden, chemosensitivity, and recurrence risk [23,24]. Other reports emphasize CEA or composite risk scores [13,25], highlighting heterogeneity across cohorts. Taken together, our findings support CA19-9 as a potentially informative prognostic marker in surgically treated RAS-wild-type mCRC, while underscoring the need for cautious interpretation and external validation.

Our study also extends the literature on metastasectomy specifically within RAS-wild-type disease. Prior work has demonstrated that RAS mutations confer inferior outcomes following metastasectomy [13,26,27]. By restricting our analysis to RAS-wild-type tumors, we show that substantial survival differences persist within this molecularly favorable subgroup, and that additional prognostic stratification may be achieved using simple biochemical markers rather than molecular status alone.

Interestingly, variables such as primary tumor sidedness and MSI status—strong predictors of response to anti-EGFR therapy and immunotherapy in advanced disease—were not independently associated with OS in the metastasectomy cohort. This observation suggests that, after surgical selection and multimodal treatment, prognostic relevance may shift away from predictive molecular features toward markers reflecting residual disease burden and micrometastatic activity.

The Turkish and regional context of our dataset deserves emphasis. Earlier national studies reported the feasibility of liver and lung metastasectomy but were often limited by single-centre design or incomplete molecular annotation [16,17]. By leveraging the ONKO-KOLON Türkiye registry, our study provides a multicentre, real-world evaluation reflecting contemporary national practice patterns. The heterogeneity in timing of metastasectomy and the frequent use of multiple systemic therapy lines underscore that these outcomes arise from integrated, multimodal care rather than protocol-driven surgical strategies, enhancing the generalizability of our findings.

Several limitations should be acknowledged. The retrospective design, missing data for some clinicopathologic variables, and limited event numbers within the metastasectomy subgroup constrained the robustness of multivariable modeling and precluded definitive causal inference. In addition, time-related and selection biases are inherent to real-world surgical cohorts and may partly explain the magnitude of observed survival differences. Nevertheless, our results are consistent with existing evidence that carefully selected patients with RAS-wild-type mCRC can experience prolonged survival when metastasectomy is incorporated into a multidisciplinary treatment strategy. Prospective studies integrating molecular profiling, treatment-response dynamics, and postoperative biomarkers such as ctDNA are needed to refine patient selection and validate prognostic models in this setting.

## Figures and Tables

**Table 1:** Baseline Demographic and Clinicopathologic Characteristics of the Metastasectomy and Non-Metastasectomy Cohorts.

Variables	Metastasectomy (*n* = 185)	No Metastasectomy (*n* = 894)	*p*-Value
**Age, years** (median [IQR])	62.0 (IQR 11)	60.5 (IQR 15)	0.084
**BMI, kg/m^2^** (median [IQR])	26.35 (IQR 6.27)	25.48 (IQR 7.13)	0.689
**Sex**			0.799
Female	62 (33.5%)	308 (34.5%)	
Male	123 (66.5%)	585 (65.5%)	
Missing	0 (0.0%)	1 (0.1%)	
**ECOG performance status**			0.103
ECOG 0–1	111 (60.0%)	473 (52.9%)	
ECOG 2–4	5 (2.7%)	46 (5.1%)	
Missing	69 (37.3%)	375 (41.9%)	
**Smoking status**			0.206
Current smoker	27 (14.6%)	159 (17.8%)	
Former smoker	27 (14.6%)	102 (11.4%)	
Never smoker	45 (24.3%)	265 (29.6%)	
Missing	86 (46.5%)	368 (41.2%)	
**Primary tumor location**			0.153
Rectum	62 (33.5%)	326 (36.5%)	
Left colon	91 (49.2%)	373 (41.7%)	
Right colon	30 (16.2%)	183 (20.5%)	
Missing	2 (1.1%)	12 (1.3%)	
**Histological grade**			**<0.001**
Grade 1	18 (9.7%)	65 (7.3%)	
Grade 2	84 (45.4%)	249 (27.9%)	
Grade 3	17 (9.2%)	77 (8.6%)	
Missing	66 (35.7%)	503 (56.3%)	
**Mucinous component**			**<0.001**
Non-mucinous	74 (40.0%)	264 (29.5%)	
Mucinous	23 (12.4%)	48 (5.4%)	
Missing	88 (47.6%)	582 (65.1%)	
**MSI status**			**<0.001**
MSI-H	16 (8.6%)	45 (5.0%)	
MSS	66 (35.7%)	213 (23.8%)	
Missing	103 (55.7%)	636 (71.1%)	
**BRAF mutation status**			0.570
Mutant	2 (1.1%)	18 (2.0%)	
Wild type	114 (61.6%)	524 (58.6%)	
Not tested	69 (37.3%)	352 (39.4%)	
**Site of metastasis**			**0.001**
Liver	157 (84.9%)	603 (67.4%)	
Lung	6 (3.2%)	74 (8.3%)	
Lymph node	6 (3.2%)	80 (8.9%)	
Peritoneal	10 (5.4%)	78 (8.7%)	
Other	6 (3.2%)	57 (6.4%)	
**Number of metastatic organs**			0.164
1	124 (67.0%)	530 (59.3%)	
2	50 (27.0%)	272 (30.4%)	
3	10 (5.4%)	79 (8.8%)	
4	1 (0.5%)	12 (1.3%)	
Missing	0 (0.0%)	1 (0.1%)	
**Liver-only metastasis**			**<0.001**
Yes	102 (55.1%)	321 (35.9%)	
No	83 (44.9%)	573 (64.1%)	
**Number of systemic therapy lines received**			0.084
0	0 (0.0%)	2 (0.2%)	
1	5 (2.7%)	12 (1.3%)	
2	64 (34.6%)	343 (38.4%)	
3	53 (28.6%)	309 (34.6%)	
4	44 (23.8%)	144 (16.1%)	
5	19 (10.3%)	84 (9.4%)	
**Baseline CEA, ng/mL** (median [IQR])	32.0 (IQR 107)	77.0 (IQR 261)	**<0.001**
**Baseline CA19-9, U/mL** (median [IQR])	25.0 (IQR 73)	38.5 (IQR 202)	**0.047**

**Footnotes**: Data are presented as median (interquartile range [IQR]) or n (%). *p*-values were calculated using the Mann–Whitney U test for continuous variables and the χ^2^ test or Fisher’s exact test for categorical variables, as appropriate. ECOG, Eastern Cooperative Oncology Group; MSI, microsatellite instability; MSI-H, microsatellite instability–high; MSS, microsatellite stable; CEA, carcinoembryonic antigen; CA19-9, carbohydrate antigen 19-9.

**Table 2:** Clinical, Pathologic, and Treatment Characteristics of the Metastasectomy Cohort (*n* = 185).

Section	Characteristic	Category	n	%
	Number of patients		185	
Metastatic disease	Type of metastatic presentation	Synchronous metastatic disease	136	73.5
		Metachronous metastatic disease	49	26.5
	Main metastatic site at diagnosis	Liver	157	84.9
		Peritoneum	10	5.4
		Lung	6	3.2
		Lymph node	6	3.2
		Adrenal	1	0.5
		Brain	2	1.1
		Other	3	1.6
Metastasectomy details	Timing of metastasectomy	Synchronous/upfront (de novo or synchronous resection)	128	69.2
		Metachronous (after primary tumor surgery)	49	26.5
		After systemic therapy	8	4.3
	Site of metastasectomy	Liver	145	78.4
		Missing	20	10.8
		Lung	12	6.5
		Lymph node	4	2.2
		Brain	2	1.1
		Bone	1	0.5
		Adrenal	1	0.5
	Number of metastatic organs at metastasectomy	1	124	67.0
		2	50	27.0
		3	10	5.4
		4	1	0.5

**Footnotes:** Clinical, pathologic, and treatment characteristics of patients undergoing metastasectomy. Percentages are calculated based on the total metastasectomy cohort (*n* = 185). Synchronous metastasectomy includes de novo or simultaneous resection of metastatic lesions, whereas metachronous metastasectomy refers to resection performed after initial treatment of the primary tumor. Percentages may not total 100% due to rounding.

**Table 3:** Univariate Cox Regression for Overall Survival in the Metastasectomy Cohort.

Variable	Comparison/Unit	HR	95% CI	*p*-Value	N Used	Events
Sex	Female vs. Male	0.571	0.320–1.021	0.059	180	58
Age	Per 1 year	1.006	0.981–1.032	0.638	179	58
Smoking status	Per 1 category increase	1.112	0.714–1.731	0.638	96	32
ECOG category	ECOG 0–1 vs. ECOG 2–4	1.397	0.191–10.243	0.742	113	34
Number of metastatic organs	Per 1 organ	0.937	0.620–1.415	0.756	180	58
Liver-only metastasis	Yes vs. No	1.027	0.613–1.723	0.919	180	58
Primary sidedness	Right vs. Left	1.547	0.791–3.026	0.202	178	56
Mucinous histology	Mucinous vs. Non-mucinous	1.098	0.454–2.654	0.835	96	25
MSI status	MSI-H vs. MSS	0.686	0.157–2.999	0.616	81	21
log10 CA19-9	Per 1 log10 unit	1.814	1.198–2.748	0.005	88	24
log10 CEA	Per 1 log10 unit	0.800	0.515–1.244	0.322	99	26
CEA baseline	Below vs. Above threshold	0.576	0.172–1.931	0.371	99	26
CA19-9 baseline	Below vs. Above threshold	0.370	0.158–0.862	0.021	88	24
Grade	Grade 1–2 vs. 3	3.160	0.747–13.366	0.118	118	34

## Data Availability

The data that support the findings of this study are available from the ONKO-KOLON Türkiye registry. Data may be shared upon reasonable request and subject to institutional approval.
